# Abdominal obesity and metabolic syndrome: exercise as medicine?

**DOI:** 10.1186/s13102-018-0097-1

**Published:** 2018-05-04

**Authors:** Carole A. Paley, Mark I. Johnson

**Affiliations:** 1 Research & Development (Ward 12), Airedale NHS Foundation Trust, Skipton Road, Steeton, Keighley, West Yorkshire BD20 6TD UK; 2 School of Clinical and Applied Sciences, Leeds Beckett University, Portland Building, City Campus, Leeds, LS1 3HE UK

**Keywords:** Metabolic syndrome, Exercise medicine, Abdominal obesity, Adiposopathy

## Abstract

**Background:**

Metabolic syndrome is defined as a cluster of at least three out of five clinical risk factors: abdominal (visceral) obesity, hypertension, elevated serum triglycerides, low serum high-density lipoprotein (HDL) and insulin resistance. It is estimated to affect over 20% of the global adult population. Abdominal (visceral) obesity is thought to be the predominant risk factor for metabolic syndrome and as predictions estimate that 50% of adults will be classified as obese by 2030 it is likely that metabolic syndrome will be a significant problem for health services and a drain on health economies.

Evidence shows that regular and consistent exercise reduces abdominal obesity and results in favourable changes in body composition. It has therefore been suggested that exercise is a medicine in its own right and should be prescribed as such.

**Purpose of this review:**

This review provides a summary of the current evidence on the pathophysiology of dysfunctional adipose tissue (adiposopathy). It describes the relationship of adiposopathy to metabolic syndrome and how exercise may mediate these processes, and evaluates current evidence on the clinical efficacy of exercise in the management of abdominal obesity. The review also discusses the type and dose of exercise needed for optimal improvements in health status in relation to the available evidence and considers the difficulty in achieving adherence to exercise programmes.

**Conclusion:**

There is moderate evidence supporting the use of programmes of exercise to reverse metabolic syndrome although at present the optimal dose and type of exercise is unknown. The main challenge for health care professionals is how to motivate individuals to participate and adherence to programmes of exercise used prophylactically and as a treatment for metabolic syndrome.

## Background

Metabolic syndrome is defined as a cluster of at least three out of five clinical risk factors: abdominal (visceral) obesity, hypertension, elevated serum triglycerides, low serum high-density lipoprotein (HDL) and insulin resistance [1]. The prevalence of metabolic syndrome has been estimated to be more than 20% of the global adult population [2, 3]. Of the five clinical risk factors used as diagnostic criteria for metabolic syndrome, abdominal obesity appears to be the most predominant [3, 4]. Obesity is defined as a body mass index (BMI) of 30 or above and has been described as a global pandemic with approximately 50% of adults worldwide expected to be obese by 2030 [5]. Abdominal (visceral) obesity, irrespective of other fat deposits, is a major risk factor for systemic inflammation, hyperlipidaemia, insulin resistance and cardiovascular disease (for review, see [6]). The role of abdominal obesity in the development of insulin resistance and the metabolic syndrome was described in 1991 [7]. However, abdominal obesity does not always occur in individuals with an elevated BMI. It was recognised as early as 1981 that normal weight, metabolically obese, individuals existed due to the presence of excessive visceral fat deposits [8].

Evidence shows that one of the single most important lifestyle changes for the prevention of many chronic diseases is exercise [9] and as a consequence exercise is now recognised as a medical treatment in its own right [6]. There is growing evidence that regular and consistent programmes of exercise will reduce abdominal fat deposits significantly, independent of weight loss [10, 11]. It is recognised that changes in body composition - particularly a reduction in abdominal fat deposits - are more important than reductions in overall body weight, or BMI, in treating metabolic syndrome. Reductions in abdominal fat deposits are important because abdominal obesity is a marker of dysfunctional adipose tissue (adiposopathy) [12]. Abdominal, or visceral obesity has a central role in the development of a pro-inflammatory state which we now know is associated with metabolic syndrome [13]. It has been suggested that exercise as a medical intervention should be prescribed in terms of its dose, i.e. mode, intensity, frequency and duration [14]. This was the basis of the American College of Sports Medicine Exercise is Medicine® (EIM) initiative [15] and their guidance on prescribing exercise [16]. As a medical intervention the prescription for exercise should also be specifically based on the individual’s capabilities and needs.

The aim of this review is to (i) summarise current evidence on the pathophysiology of dysfunctional adipose tissue (adiposopathy), its relationship to metabolic syndrome and how exercise may mediate these processes; and (ii) evaluate current evidence on the clinical efficacy of exercise in the management of abdominal obesity and to assess the type and dose of exercise needed for optimal improvements in health status.

### Abdominal obesity, adiposopathy and metabolic dysfunction

To understand the significance of abdominal obesity and its contribution to metabolic syndrome, it is necessary to appreciate the link between the diseases associated with this condition. The accumulation of ectopic fat in tissue surrounding the viscera is directly related to the development of insulin resistance [17]. Insulin resistance is thought to be the common denominator in the development of metabolic syndrome. In addition, evidence suggests that systemic inflammation is an important factor in its development, through the development of insulin resistance [18–21]. Visceral fat deposits (abdominal adiposity) are associated with the development of adipose cells that are enlarged and dysfunctional (adiposopathy, or ‘sick fat’) [21]. Dysfunctional adipose tissue secretes pro-inflammatory biomarkers including prostaglandins, C-reactive protein (CRP), and cytokines such as interleukins (e.g. interleukin-6), tumour necrosis factor alpha (TNF-α), and leptin [22, 23]. With increasing obesity there is also a corresponding decrease in levels of adiponectin, an antiatherosclerotic adipokine [24]. Inflammatory mediators released by adipose tissue contribute to the development of type II diabetes, hyperlipidaemia and cardiovascular disease [25, 26]. If there is a high proportion of fat to muscle this is likely to contribute to this metabolic dysfunction as an increase in circulation of free fatty acids requires greater insulin secretion for control of glucose metabolism. The resulting hyperinsulinaemia desensitises insulin-sensitive tissues, which predisposes individuals to type II diabetes [27]. The decrease in adiponectin secretion also inhibits insulin receptor proteins. Moreover, regular consumption of foods rich in carbohydrate results in postprandial hyperglycaemia which causes repetitive acute inflammation which might contribute to a chronic inflammatory state [28]. Chronic systemic inflammation increases oxidative stress and reduces metabolic flexibility, thus perpetuating metabolic syndrome, leading to a vicious cycle of disease, depression and further inactivity [29, 30].

Adipose tissue hypoxia also occurs in the obese state although the mechanisms for this are not fully understood [19]. It has been suggested that deficient angiogenesis causes decreased blood flow due to reduction in capillary density and excessive growth of adipose tissue. This may also be exacerbated by obstructive sleep apnoea which is common in obese individuals, and results in a reduction of oxygen to the tissues [31]. Adipose tissue, hypoxia is associated with an increased expression of inflammatory genes and decreased expression of adiponectin, resulting in local and systemic inflammation [19, 32, 33]. The response to adipose tissue hypoxia includes insulin sensitivity and glucose intolerance as adiponectin is associated with normal glucose and lipid metabolism. Leptin expression has also been shown to increase in obesity and the likely explanation for this is adipose tissue hypoxia [34]. This is important as leptin expression modulates insulin resistance [35]. Furthermore, ghrelin regulation in obese individuals is affected and serum ghrelin suppression in response to stomach fullness is impaired which results in a failure to suppress the continued desire to eat, thus compounding the problem [35].

Hypothlamic-pituitary-adrenal (HPA) axis hyperactivity is evident in abdominal obesity and is also associated with insulin resistance due to an increase in cortisol levels [36]. Cortisol, secreted by the adrenal glands, is involved in glucogenesis which increases blood sugar as a response to stress. Epidemiological data provide evidence for a significant positive association between increased cortisol levels and the risk of developing type II diabetes and atherosclerosis due to a failure to suppress inflammation [37]. Also, the secretion of low grade inflammatory mediators by adipose tissues may act as an additional chronic stimulus to the activation of the HPA axis which in turn results in increased levels of cortisol secretion, resulting in a positive feedback loop [38].

It is important to note that not all obese patients develop metabolic syndrome and there exists a cohort of metabolically ‘healthy’ individuals who are obese [39]. At present, there is no explanation for this and it is not known whether these metabolically healthy obese individuals will eventually develop metabolic syndrome and are simply experiencing a delayed-onset of disease [24]. Interestingly, there are also normal weight individuals who are regarded as ‘metabolically obese’ due to the storage of ectopic fat around the viscera whilst maintaining a normal BMI [40, 41].

When BMI is used as a measure of obesity only a modest association with cardiovascular risk factors is found [18]. However, when abdominal obesity measurements, such as waist circumference or waist:hip ratio are included as a measure of abdominal adiposity a strong association with cardiovascular and metabolic syndrome risk factors is found [42–45].

### Metabolic dysfunction and exercise

Abdominal adiposity is a reversible condition and its reduction can have excellent effects in diminishing cardiovascular and metabolic syndrome risk. Evidence from a study by Brooks, et al. demonstrated that increased abdominal obesity was associated with systemic inflammation as measured by high-sensitivity C-reactive protein (hsCRP) [18]. Given the direct link between abdominal obesity and systemic inflammation it is not surprising that even modest reductions in abdominal adipose tissue are accompanied by improvements in metabolic function and reduced cardiovascular risk.

Several studies show a strong association between obesity and physical inactivity [46–48] and that metabolic syndrome is associated with sedentary lifestyle and poor cardiorespiratory fitness [49]. Sedentary behaviour is widely regarded as activity which involves energy expenditure at the level of 1.0–1.5 metabolic equivalent units (METS),which usually involves time sitting or lying down and includes office and computer work and watching television [50]. Edwardson et al. conducted a meta-analysis that found that individuals who spend more time in sedentary behaviours have greater odds of having metabolic syndrome [50]. A prospective study which examined the relationship between sedentary behavior and metabolic syndrome in 930 men found that men with middle and high sedentary behavior had a higher risk of developing metabolic syndrome (65% middle and 76% high level sedentary behaviour respectively), than men who were active [51]. A longitudinal study observing 4840 adults found that improvements in cardiometabolic factors occurred in overweight and obese individuals with increased levels of physical activity, although the participants were those participating in a health screening programme and were therefore probably of a higher economic status. At follow-up, there was a statistically significant decrease in non-HDL concentrations of 5.8% (overweight) and 4.6% (obese) relative to baseline, and a decrease in low-density lipoprotein (LDL) cholesterol concentrations of 4.7% (overweight) and 6.1% (obese) relative to baseline [52]. Of the parameters observed, non-HDL cholesterol and plasma triglycerides were found to have the largest improvement when physical activity was increased. A study followed 22,383 participants, aged 30–64 years, comparing metabolic syndrome risk with intensity level of leisure-time exercise and by occupational and commuting activity [53]. Leisure-time activity was found to be linearly and inversely associated with a risk of developing metabolic syndrome and vigorous-intensity activity alone or a combination of both moderate- and vigorous-intensity activity was associated with a lower risk of metabolic syndrome. The researchers classified activity levels according to the MET equivalent: moderate-intensity at 3–6 METs and vigorous-intensity > 6 METs. The introduction of increased physical activity into a previously inactive lifestyle might also break the cycle of inflammation-mediated sickness behaviour as described by Nunn, which suppresses the desire to undertake physical activity [30].

A systematic review and meta-analysis was conducted by Ostman et al. 2017 to determine whether exercise reversed various indices of metabolic syndrome including body composition, blood cholesterol, fasting blood glucose, fasting insulin, blood pressure and clinical outcome [54]. A total of 16 studies (800 participants) were included in the review and it was found that aerobic training produced small improvements in fasting blood glucose, triglycerides and low-density lipoproteins. Combined aerobic and resistance exercise training resulted a reduction of 13% in triglycerides only. Nevertheless, combined with improvements in maximal oxygen uptake and blood pressure, the overall risk profile for patients was much improved. When the combined exercise group was compared with the control group the mean difference of: waist circumference was − 3.80 cm (95% CI − 5.65, − 1.95, *p* < 0.0001); systolic blood pressure was − 3.79 mmHg (95% CI − 6.18, − 1.40, *p* = 0.002); and HDL was 0.14 (95% CI 0.04, 0.25, *p* = 0.009). The improvements in waist measurement would suggest that the long-term risks associated with metabolic syndrome were reduced. An earlier review to determine the effectiveness of ‘lifestyle modification programmes’ on improving metabolic risk factors (blood pressure, triglycerides and waist circumference) in adults with metabolic syndrome found reductions in these measures, although such programmes were said to be more effective if carried out for more than 12 weeks, thus emphasising the need for long-term lifestyle modifications [55].

There are a number of studies which have specifically investigated the effect of exercise on abdominal obesity, irrespective of total body weight and these are summarised in a comprehensive review by Pedersen and Saltin [56]. Amongst their findings they reported that a cross-sectional study of overweight males showed that those with a high level of fitness (as measured by activity and maximal oxygen uptake) had lower levels of visceral fat than their unfit counterparts when scanned using magnetic resonance imaging [39]. Lee et al. investigated the effects of exercise without weight loss on total and abdominal adiposity and skeletal muscle mass and composition in previously sedentary, lean men and in obese men with and without type II diabetes [11]. It was found that, even in the absence of weight loss, moderate-intensity exercise was associated with significant reductions in total and abdominal fat, and there was a reduction in skeletal muscle lipid content independent of group. Stewart et al. investigated the effects of exercise on cardiovascular and metabolic disease in older adults and found that reductions in total and abdominal fatness and increase in leanness were strongly associated with reductions in risk factors for cardiovascular disease and diabetes, including those that constitute metabolic syndrome [57]. Lee et al. conducted a longitudinal study of 32,593 adults who underwent an abdominal computerised tomography scan as part of health screening and found that the ratio of visceral-to-subcutaneous fat was independently associated with all-cause mortality. This suggests that the location of fat deposits in the abdomen (viscera) is a better indicator of metabolic risk than total body fat, which is unsurprising given the positive association between abdominal adiposity and systemic inflammation [58].

A number of reviews have shown that exercise training specifically elicits an anti-inflammatory effect, independent of weight loss [33, 59–62]. Other metabolic benefits of exercise were reported in a study on patients with type II diabetes where pedometer-measured exercise was not only associated with reductions in systemic inflammation, but also reductions in abdominal obesity and arterial stiffness [63]. One of the mechanisms for the anti-inflammatory effect of exercise is a reduction in adipose tissue hypoxia resulting from improved capillary density blood flow. In a review by Golbidi [24] the inverse relationship between exercise, body mass index (BMI), hip-waist ratio, and waist circumference was described. The anti-inflammatory effect of exercise was also explained as being closely related to oxidative stress. Exercise was shown to improve glucose tolerance, insulin resistance and lipid metabolism and reduce blood pressure in both healthy individuals and those with metabolic disease. Large population cohort studies observed relationships between plasma CRP and the level of exercise that was independent of obesity as measured by body mass index [62, 64]. The effect of exercise training on CRP was investigated in a systematic review which considered a total of 83 studies of different types. It was found that exercise training led to a greater reduction in CRP when accompanied by a decrease in BMI, but that significant reductions in CRP occurred without weight loss [65]. Furthermore, a Cochrane review provided evidence that exercise improved general health even where no weight was lost because it improved plasma lipoprotein profile [66].

Not all studies provide evidence that exercise training reduces pro-inflammatory biomarkers. Melo et al. reviewed 11 studies of patients with type II diabetes and found insufficient evidence to determine whether aerobic or resistance exercise improved systemic levels of inflammatory markers [67]. However, an earlier review by Hayashino et al. found that both CRP and IL-6 were reduced by exercise training [68]. It is still unclear whether improvements in inflammatory status are independent of weight loss or entirely dependent upon the changes in body composition that result from exercise training [61]. Nevertheless, Eaton and Eaton observed that the percentage of lean body mass is critical in avoiding the hyperinsulinaemia which predisposes individuals to type II diabetes because a greater insulin secretion is required for any given glucose load where levels of body fat are disproportionate [27]. This would suggest that strength training that develops lean tissue is critical in the treatment, or prevention, of metabolic disease.

### Optimal dose of exercise

There are no specific guidelines on exercise prescription for systemic inflammation although guidance is available in the form of programmes designed to reduce body fat and improve general health status. The American College of Sports Medicine (ACSM) recommends 150–250 min of moderate-intensity exercise per week as optimal but other authors have suggested between 30 [69] and 60 [70] minutes per day would be required. There is a consensus that performing 3000 steps (~ 30 min of activity) per day over and above normal activities is sufficient for improvements in health status but perhaps not optimal according to the ACSM recommendations [71–75]. A systematic review and meta-analysis by Hayashino et al. [68] was conducted to assess the effects of exercise interventions on inflammatory markers/cytokines and adipokines which contribute to the development of atherosclerosis, insulin resistance, and development of late-onset complication in patients with type II diabetes. They found that exercise training with a longer duration and frequency was more effective in reducing systemic inflammation, suggesting that these effects might be dose-dependent. More recently, this idea has been challenged and it is now thought that shorter-duration, higher intensity interval training (HIIT) is beneficial [76]. Recent findings suggest that HIIT programmes are effective in reducing metabolic syndrome combined with high adherence rates and this is important because incorporating HIIT programmes into daily life is less disruptive. Gremeaux, et al. studied the effects of HIIT training on a sample of 62 overweight or obese adults who were above the recommended abdominal obesity threshold. All participants completed 2–3 weekly sessions of repeated short-duration (15–30 s) interval training at 80% of their aerobic threshold. It was found that the prevalence of metabolic syndrome was reduced by 32.5% at the 9 month follow-up. Importantly, adherence rates to the programme were 97%.

In a study designed to evaluate the effects of different intensity exercise programmes combined with a healthy diet in subjects with metabolic syndrome, 75 non-diabetic subjects were recruited to undertake either a programme of 10,000 steps per day, a fitness programme involving activity at > 75% peak VO_(2)_ three times per week or a 12 week programme of walking 1 h each day [77]. The metabolic and vascular effects of these three different regimens were studied and improvements were observed in various measures including BMI, waist measurement, glucose metabolism, insulin resistance and lipid profiles. The more intense exercise regimen at > 75% peak VO_2_, combined with a low-sugar diet, was most effective, which provides further support to the evidence showing the benefits of HIIT training in combination with dietary advice. A significant observation was that in 64% of the study participants metabolic syndrome was resolved.

Zhang et al. also found that high intensity interval training was better than continuous moderate aerobic training in reducing abdominal visceral fat in obese young women [78]. Similar findings from other studies support the benefit of high-intensity interval training performed in short, high-intensity bursts, involving as little as 10 min of activity at a time, and this might promote better adherence in non-habitual exercisers [79–81]. A further study of 2330 adults found that consistent moderate to vigorous activity was more important than exercise volume in reducing CRP levels associated with systemic inflammation [82]. A systematic review by Cronin et al. found that greater reductions in inflammatory biomarkers occurred in older healthy inactive participants when higher intensity aerobic exercise was undertaken [83].

A review by Zdziarski et al. found that largest reductions in systemic inflammation and improvements in well-being, depression and sleep was achieved using multi-modal exercise (aerobic and resistance training) in individuals with inflammation-related chronic pain [84]. This is important because it is likely that individuals in a pro-inflammatory state due to abdominal adiposopathy may also be susceptible to chronic pain conditions. Dutheil et al. reported that high resistance-moderate endurance training was efficient in improving visceral fat loss in 100 healthy adults [85]. If changes in body composition are more important than total body weight loss then resistance training combined with aerobic exercise would produce optimal effects in increasing percentage lean body mass [27].

### Promoting adherence to exercise Programmes

One of the major challenges in using programmes of exercise to improve health status is promoting and maintaining adherence in individuals who have often been inactive for many years and who may be overweight or obese [86]. Ideally, therefore, attempts should be made to include exercise into normal daily life although attrition rates can still be as high as 50% [87]. To promote adherence Clauw and Crofford suggested that additional activity is incorporated very gradually – as little as 5 min daily [88] although the programme needs to be tailored to the individual whilst aiming to deliver optimal effects [84]. As discussed above, the recent findings that HIIT programmes are effective in reducing metabolic syndrome combined with high adherence rates is significant because incorporating it into daily life is less disruptive. Connelly et al. conducted a review to assess the effectiveness of technology to promote physical activity in people with Type 2 diabetes and found that the use of technology-based interventions, such as mobile phone applications, texts and email support, improves compliance [89].

In summary, evidence suggests that optimal abdominal fat reduction and the development of lean tissue is achieved by combining high-intensity interval training and resistance training with an overall general increase in daily physical activity.

## Conclusion

An increasingly sedentary lifestyle, a lack of regular exercise and an increase in obesity have been the main contributors to a rise in the incidence of metabolic dysfunction, particularly in the developed world. There is moderate evidence supporting the use of programmes of exercise to reverse metabolic syndrome although at present the optimal dose and type of exercise is unknown. The main challenge for health care professionals is how to motivate individuals to participate and adherence to programmes of exercise used prophylactically and as a treatment for metabolic syndrome.

## Abbreviations

ACSM: American College of Sports Medicine
BMI: Body mass index
CRP: C-reactive protein
EIM: Exercise is Medicine
HDL: High density lipoprotein
HIIT: High Intensity Interval Training
HPA: Hypothlamic-pituitary-adrenal
hsCRP: High-sensitivity C-reactive protein
LDL: Low density lipoprotein
METS: Metabolic equivalent units
TNF-α: Tumour necrosis factor alpha
VO_2_: Oxygen Uptake
